# Metabolic expenditure, neurodevelopment, and weight gain into early childhood after fetal growth restriction

**DOI:** 10.1038/s41598-026-53713-y

**Published:** 2026-06-15

**Authors:** Cigdem Gelegen, Beatrice Copley, Neelum Mistry, Chiara Sacchi, Chiara Nosarti, Lorenzo Fabrizi, Anna L. David, Kimberley Whitehead

**Affiliations:** 1 Elizabeth Garrett Anderson Institute for Women’s Health, University College London, London, WC1E 6AU UK; 2 Research Division of Digital Health and Applied Technology Assessment (DHATA), King’s College London, London, SE1 8WA UK; 3 Department of Neuroscience, Physiology and Pharmacology, University College London, London, WC1E 6BT UK; 4 Department of Developmental Psychology and Socialisation, University of Padua, Padua, 35122 Italy; 5 Department of Early Life Imaging, School of Biomedical Engineering and Imaging Sciences, King’s College London, London, SE1 7EH UK; 6 Department of Child & Adolescent Psychiatry, King’s College London, London, SE1 7EH UK

**Keywords:** Autonomic, Developmental origins of health and disease, Intrauterine growth restriction, Placental insufficiency, Prematurity, Neonatal, Medical research, Neuroscience, Physiology

## Abstract

Fetal growth restriction (FGR) subjects exhibit altered metabolism, with higher metabolic rate due to their small body mass, and by adopting strategies to minimise energy expenditure. We investigated how these metabolic differences develop, or manifest in growth trajectories, after FGR, small for gestational age (SGA) with no evidence of FGR, and normal pregnancies. We curated a unique composite dataset of subjects between 14 weeks of gestation and six years of age. First, we assessed fetal and infant heart rate to assess whether higher metabolic rate persisted postnatally after FGR. Next, as the largest energy expenditure is brain synaptic maintenance, we tested whether FGR infants had lower white matter volume (proxy for synapse number). Finally, we modelled longitudinal body weight into childhood in FGR, SGA, and control groups, and tested for associations with neurodevelopmental scores at 1–2 years. Heart rate at rest was 3.2 beats per minute higher in FGR fetuses and infants (710 subjects), and FGR infants exhibited only 52% the increase in heart rate to a nociceptive procedure (i.e. a physiological challenge) observed in controls. FGR infants had 7 cm^3^ smaller white matter volume (270 subjects). Finally, for every − 100 g an individual’s weight deviated below their group’s average (1660 subjects), their motor score was 0.4 points lower (1030 subjects). FGR subjects continue to exhibit metabolic differences long after birth, which may further disadvantage them beyond the initial antenatal insult. Growth trajectories encode information about how FGR is transmitted into suboptimal neurodevelopment, and could identify intervention opportunities.

**Supplementary Information:**

The online version contains supplementary material available at 10.1038/s41598-026-53713-y.

## Introduction

Early growth is a metabolically expensive process^1–3^, with a dramatic peak in metabolic rate to 6 months of age^4^. The energy-demanding brain is a major driver, particularly as it occupies a larger proportion of total body mass in young children^5–7^. Whole-body metabolic rate can be inferred from surrogate metrics which index resource supply, including heart rate^8–10^, allowing data to be modelled through gestation when direct measurements of metabolism are impossible.

Antenatally, when metabolic demands are not met by placentally-mediated resources, the result is fetal growth restriction (FGR): a condition that is a risk factor for stillbirth^11^. The vast majority of FGR subjects are small for gestational age (SGA) (< 10th weight centile). However, the inverse is not true: only a subset of SGA subjects are FGR, with the remainder constitutionally small (i.e. in accordance with their genetic growth potential)^12^. Especially when FGR is dissociated from the larger SGA population, FGR predicts adverse neurodevelopmental and metabolic outcomes in survivors both in the short-term and long-term into adulthood^11^.

Whole-body metabolic rate is higher in FGR^13–15^, likely owing to their lower body mass and that more of this mass is occupied by the brain^16 ^(for the underlying biomathematics, please see^17,18)^. In line with this, higher heart rates are observed in FGR fetuses^19–22^, and FGR neonates on the first postnatal day^23,24^. However, it is unknown how heart rate differences develop postnatally, during the crucial first extrauterine weeks after a growth-restricted pregnancy. This is especially important because higher basal heart rate means that this organ is already functioning closer to its upper limit, which may impair autonomic heart rate reactivity to a physiological challenge^8,25^. To address these questions here, we examined both resting heart rate, and its reactivity.

Being solely dependent on fetoplacental nutrient and oxygen exchange to fulfil metabolic demands, FGR fetuses adopt mechanisms to minimise energy expenditure and facilitate growth, including reduced movements and reduced rapid eye movement sleep^26–28^. A huge energy expenditure during early development is the maintenance of synapses, including the spontaneous electrical activity that sustains them^29,30^. Post-mortem data demonstrate a decreased number of synapses after FGR^31–33^. Given that a proxy for synapse number is white matter volume^17^, we tested here whether neonates with FGR had lower white matter volume, which would indicate efforts to reduce brain-related energy costs.

Finally, the degree to which energy costs are met by metabolic rates can be assessed longitudinally using body weight growth trajectories: poor growth indicates a shortfall. Slower fetal growth and lower birth weight predict later adverse neurodevelopment^34^. However, such early measurement windows miss the crucial metabolically-demanding postnatal growth phase to 6 months^4^. This is when growth falters the most in another vulnerable group (infants living in countries with high rates of undernutrition)^35^. Here we tracked longitudinal fetal-postnatal growth from 14 weeks since conception to six years of age, and assessed whether shortfalls in growth were associated with lower neurodevelopmental scores.

## Methods

### Data

A composite dataset was created. We merged data collected at University College London Hospitals (UCLH) within a neurophysiological research programme^36,37^, with the Europe-wide (including UCLH) EVERREST cohort^38^, the Evaluation of Preterm Imaging Study (ePrime)^39^, and open-access data from FEMINA2^27^, the IEEE dataport^40^, and the Norway-Alabama Fetal Growth Study^41–44^. Of the three open-access datasets, the first two are freely available, while the Norway-Alabama dataset is obtainable upon written application to the Biospecimen Repository Access and Data Sharing (BRADS) committee, Division of Intramural Population Health Research (DIPHR)^45^, and permissions were granted to KW in 2023. The neurophysiological, ePrime, and FEMINA2 datasets included FGR and SGA subjects, but were not set up to address these populations specifically. The EVERREST, IEEE, and Norway-Alabama datasets were set up to research the FGR population. Overall then, this composite dataset was enriched for FGR subjects.

For all studies, ethical approval to conduct the work was secured from the relevant body, informed written parental consent was provided, and research was performed in accordance with relevant guidelines and regulations including the Declaration of Helsinki. The details of ethical approval for our own (not open-access) data are: UCLH neurophysiological: London-Surrey borders Research Ethics Committee (REC) (reference: 11/LO/0350); EVERREST: London-Stanmore REC in England (13/LO/1254), Hospital Clinic of Barcelona’s Clinical REC in Spain (HCB/2014/0091), Regional Ethical Review Board in Sweden (DNr 2014/147), and Ethics Committee of the Hamburg Board of Physicians in Germany (PV4809); ePrime: Hammersmith and Queen Charlotte’s REC (09/H0707/98).

### Subjects

Being small for gestational age (SGA) and sex, defined as < 10th birth weight centile^46^, is a risk factor for adverse outcomes, with risk increasing when FGR is dissociated from the larger SGA population^34,47–50^. In FGR, the fetus is smaller than its genetic growth potential with evidence of placental insufficiency^51^. FGR can be subcategorised into early-onset (FGR-EO) when it is diagnosed before 32 weeks of gestation, which has worse outcomes compared to late-onset FGR which presents from 32 weeks^52^. Consequently, relative to (i) controls, being (ii) SGA with no evidence of FGR, (iii) FGR, or (iv) FGR-EO comprises a scale of ascending risk.

Here, controls had no evidence of SGA or FGR, and - when analysing postnatal measures - we confirmed live birth ≥ 15th weight centile and < 5000 g^53^. SGA was defined as < 10th birth weight centile, in the absence of meeting criteria for FGR. Where possible, FGR was defined according to the international Delphi consensus criteria^52^. In ePrime, FGR was defined by medical discharge records-reported abnormal fetal scans and/or Doppler ultrasound measurements, or < 10th birth weight centile combined with clinical evaluation of FGR and evidence of antenatal adversity associated with placental insufficiency (e.g. maternal preeclampsia)^49^. In the Norway-Alabama dataset, FGR was defined as < 10th birth weight centile and evidence of antenatal adversity associated with placental insufficiency (e.g. maternal preeclampsia)^45^. Given that the Norway-Alabama dataset was the only one to dissociate FGR from SGA without Doppler measurements (Table S1), we reviewed those subjects’ placental data - when available - to confirm that FGR classification was associated with markers of placental insufficiency (Supplementary).

Multiple pregnancies were eligible; classification used standard (singleton) growth charts, which have higher sensitivity but lower specificity to detect clinically significant small size in twins^54^. Therefore, FGR was only assigned in multiples when there was evidence of selective FGR^55^: this implies placental insufficiency given its association with adverse outcomes^56^. In cases when Doppler information was unavailable, this required one twin to be < 10th birth weight centile and the other not, in the presence of both (i) evidence of antenatal adversity associated with placental insufficiency, and (ii) birth weight discrepancy ≥ 18%^56^. Otherwise, the smaller twin was assigned as SGA. If all multiples of a pregnancy were ≥ 15th birth weight centile, they could enter the control category. If both twins were small, or one twin was < 10th and the other 10 < 15th birth weight centile, the pregnancy’s data were excluded.

In FGR-EO, before 32 weeks of gestation the fetus is either very small (< 3rd centile) or presents with evidence of placental insufficiency including abnormal uterine or umbilical artery Doppler measurements^52^. Here, FGR-EO could be confirmed according to these criteria in 87.4% of cases (see Table 1 footnote for details), and this subcategory was otherwise assigned to infants with FGR born very preterm (given that they must by definition have FGR-EO). As it was not possible to establish whether FGR was early-onset in some cases, subjects were assigned to one of two subcategories: FGR (no positive evidence for early-onset) and FGR-EO (positive evidence for early-onset) (Table 1).

Exclusion criteria included confirmed or suspected congenital or other non-placentally mediated cause for small fetal size (e.g. structural anomaly, aneuploidy, cytomegalovirus infection). In subjects who suffered an acute neurological insult such as hypoxic-ischemic encephalopathy, data prior to but not subsequent to the insult were eligible for analysis. Table S1 provides more details about each dataset, and Table S2 describes to which analyses they contributed.

### Heart rate at rest

Fetal heart rate at rest was sourced from that calculated during clinical cardiotocography (CTG) monitoring. Data during labour were not included. Recordings lasted for up to 45 min (FEMINA2), exactly 40 min (IEEE), or as clinically indicated (neuro and EVERREST).

Postnatal heart rate at rest was calculated from transcutaneous pulse oximetry, or ECG recordings excluding any periods of sustained crying. For ECG recordings, automated beat detection was performed using LabChart v.8 HRV software (ADInstruments, Spechbach, Germany), following which all data were visually inspected and missing beats manually added if necessary.

### Heart rate reactivity to a stimulus

In the neurophysiological research, studies had often been timed around a clinically necessary nociceptive procedure (heel lance)^37^. To examine heart rate reactivity to the nociceptive procedure, we subtracted the mean heart rate during the preceding 15 s, from the maximum heart rate across the subsequent 30 s (sampling rate: 2 s, i.e. 0.5 Hz).

### Neonatal structural MRI

MRI data analysed were from the ePrime subjects described in^49^, but our sample size was slightly smaller because we aligned inclusion/exclusion criteria to our own (e.g. that control infants must be ≥ 15th birth weight centile (the criteria in^49^ was ≥ 10th)). Acquisition details are available in^49^, and the pre-processing methodology used is described in^57^. In brief, the motion-corrected, reconstructed T2-weighted image was first bias-corrected and brain-extracted. Following this, the brain image was segmented into different tissue types using the validated Draw-EM algorithm^57^. Segments included white matter, which is analysed here. White matter volume was normalised by total intracranial volume (which included total brain volume and cerebrospinal fluid).

### Biometry

Estimated fetal body weight was calculated using ultrasound biometric measurements according to established formulae^58,59^, in pregnancies ≥ 14 weeks since conception^60–62^. Postnatally, body weight was measured directly. Table S2 details the most common developmental time points that weight was assessed across datasets.

### Bayley scales of infant and toddler development

Bayley scales are age-standardised, and the scores have a reported mean and standard deviation of 100 and 15 respectively. We analysed scores from either 1 or 2 years, utilising the later age point when scores from both ages were present. Cognitive and motor scores were assessed, because the language score was less consistently available. The Bayley scales are periodically updated and the version used differed within this composite dataset (cognitive: Mental Developmental Index or Cognitive Composite; motor: Psychomotor Developmental Index or Motor Composite). This was accounted for by using a conversion formula so that all scores were aligned to the Cognitive^63^ or Motor Composite^64^. As age-standardised Bayley scores can decline between 1 and 2 years in high-risk populations^65^, implying emerging impairment, we also adjusted in our statistical models for whether the score analysed was from 1 or 2 years.

### Statistics: regression models

Data were processed using R v.4.4^66^. To visualise data, we used local polynomial regression fitting of the means. To analyse data, we used linear models (supplemented by package effects^67,68)^. Analysis of data that followed a notably non-linear trajectory utilised piecewise regression using the bs() function within package splines (the local fitting used for visualisation does not produce a readily interpretable change curve equation). Piecewise models allow to fit pieces of a trajectory separately (divided by ‘knots’), approximating a curve^69^, and have been used successfully to model developmental trajectories^70^. Package lspline was used for extracting slopes across each piece^71^.

Repeated or clustered measures were handled with a multilevel linear model using package lme4^72^. This ensures that standard errors are valid, as the non-independence of within-unit measures is accounted for. In a multilevel model, a random effect is the unit (e.g. subject) deviation in intercept from fixed effects (regression coefficients). Up to two random effects were included: (i) subject, (ii) site (Table S1). The random effect of subject was always included in case of repeated measures. Each subject has just one random effect, irrespective of how many measures they had, as this reflects their individual regression line. For site, we reviewed its influence for each analysis: if its intraclass correlation coefficient (ICC) was > 0.00 (observations within site were more similar than between sites), we retained it as a random effect, with the subject random effects nested within if applicable. To interrogate any site effects, we re-calculated fixed effects coefficients with each site deleted in turn^73^. We treated the resultant range of coefficients similarly to confidence intervals: if the range crossed zero, we considered the fixed effect unreliable.

### Statistics: variables

In all analyses, group (FGR-EO, FGR, and SGA were compared to controls) and developmental change were the primary variables of interest, and sex was adjusted for. The other variables of interest for each analysis are described in the text. These encompassed postnatal vs. antenatal status for integrated antenatal-postnatal analyses, and weeks since conception at delivery for postnatal analyses. (In analyses including antenatal data, weeks since conception at delivery was a future event, and not always available, and therefore not included in the model.) All variables were entered into each final model together, so each effect size reported is adjusted for the effects of the other variables (reported means are estimated adjusted means^67,68)^. For figures, only raw, unadjusted values are plotted, unless otherwise stated. To assess whether the inclusion of additional variables, or interaction terms, improved model fit, we used Akaike Information Criterion (AIC), which penalises models with a higher number of parameters. Additional variables or interaction terms were retained if AIC decreased by > 4.0 units^74^, unless collinearity produced illogical coefficients. Harm produced by collinearity was measured using generalised variance-inflation factors (GVIF^1/(2x*df*)^): these adjusted factors allow to compare collinearity of independent variables^75^. Throughout, we follow an effect size approach to statistical inference, in which t or z values (the ratio of an independent variable’s coefficient divided by its standard error) larger than 2.0 are reported^69^.

### Statistics: sensitivity analysis re. classification method

As explained above, we adopted a classification method that allowed us to dissociate FGR from SGA with no evidence of FGR, and thereby order subjects along a scale of ascending risk: controls < SGA< FGR < FGR-EO. If our approach was robust, we expected that an alternative method that also stratified subjects along a biological gradient of risk would show similar results, whereas a method that did *not* dissociate FGR from SGA would show smaller effect sizes of difference. To test this, we conducted two sensitivity analyses. We re-ran models with the same variables except that the original classification method was substituted with (i) placing subjects along a continuous scale according to body weight (birth weight, or body weight +/- 2 days of the relevant measure), or (ii) binarising subjects into < 10th birth weight centile (yes/no). These sensitivity analyses were restricted to dependent variables other than body weight, to avoid circularity. Sometimes the sample size for these sensitivity analyses was smaller than the original analyses due to missing data: if so, this is reported in the text.

### Statistics: sensitivity analysis re. influence of multiple pregnancies

In multiple pregnancies, it may be more difficult to correctly identify FGR or SGA^76^. Hence, for analyses that included any multiple gestation subjects classified in the absence of Doppler information (Methods/*Subjects*), we conducted a sensitivity analysis by re-running the model with all multiple gestation subjects excluded. Otherwise, we report the percentage of multiple gestation subjects for any statistically significant result.

## Results

The demographics of the subjects are described in Table 1.

**Table 1:** Demographics.

	Total, *N* = 1922 (100%)	Control, *N* = 1166 (60.7%)	SGA with no evidence of FGR, *N* = 311 (16.2%)	FGR, *N* = 206 (10.7%)	FGR-EO ^a^, *N* = 239 (12.4%)
**Sex**					
Female	925 (48%)	546 (47%)	157 (50%)	112 (54%)	110 (46%)
Male	966 (50%)	620 (53%)	154 (50%)	94 (46%)	98 (41%)
Undetermined	31 (1.6%)	0 (0%)	0 (0%)	0 (0%)	31 (13%)
**Pregnancy**					
Singleton	1,776 (92%)	1,052 (90%)	300 (96%)	200 (97%)	224 (94%)
Multiple	146 (7.6%)	114 (9.8%)	11 (3.5%)	6 (2.9%)	15 (6.3%)
**Weight at delivery** (g) ^b^					
Mean (SD)	2,549 (974)	2,848 (971)	2,509 (351)	2,309 (417)	1,185 (596)
**Live born**					
Yes	1,879 (98%)	1,166 (100%)	311 (100%)	206 (100%)	196 (82%)
Unknown	4 (0.2%)	0 (0%)	0 (0%)	0 (0%)	4 (1.7%)
No	39 (2.0%)	0 (0%)	0 (0%)	0 (0%)	39 (16%)
**Dataset** ^c^					
Alabama	755 (39%)	437 (37%)	253 (81%)	45 (22%)	20 (8.4%)
ePrime	270 (14%)	223 (19%)	0 (0%)	0 (0%)	47 (20%)
EVERREST	133 (6.9%)	0 (0%)	0 (0%)	0 (0%)	133 (56%)
FEMINA2	277 (14%)	232 (20%)	29 (9.3%)	15 (7.3%)	1 (0.4%)
IEEE	262 (14%)	160 (14%)	0 (0%)	102 (50%)	0 (0%)
neuro	104 (5.4%)	51 (4.4%)	4 (1.3%)	23 (11%)	26 (11%)
Norway	121 (6.3%)	63 (5.4%)	25 (8.0%)	21 (10%)	12 (5.0%)

Percentages ≥ 10% are rounded to the nearest whole number.

^a^ We were able to confirm in 209/239 (87.4%) FGR-EO subjects that, before 32 weeks since conception, the estimated fetal weight (EFW) or abdominal circumference (AC) was <3rd centile (179 subjects) and/or the umbilical artery had absent end-diastolic flow or the EFW/AC was < 10th centile with abnormal uterine or umbilical artery Doppler measurements (86 subjects)^52^.

^b^ Two FGR subjects were > 10th birth weight centile (range: 13-18th); both had EFWs on the 4th centile and static fetal growth^52^. 9/196 (4.6%) live-born FGR-EO subjects were > 10th birth weight centile (range: 10-25th); all had abnormal Doppler measurements^52^.

^c^ See Table S1 and Table S2 for further details.

### Heart rate

#### FGR-EO subjects had a higher heart rate at rest

We analysed heart rate at rest between 16 and 92 weeks since conception (approximately 2 months old) (total 710 subjects, 1242 measures; FGR-EO: 142 subjects, 648 measures; FGR: 132 subjects, 148 measures; SGA: 28 subjects (no repeated measures); controls: 408 subjects, 418 measures). Data for the period between 16 and 26 weeks were only available for the FGR-EO group. The overall proportions of antenatal: postnatal measures were 1047:195 (84%:16%) (similar proportion of antenatal across groups: 80–90%). Heart rate was not associated with study site.

**Fig. 1:**
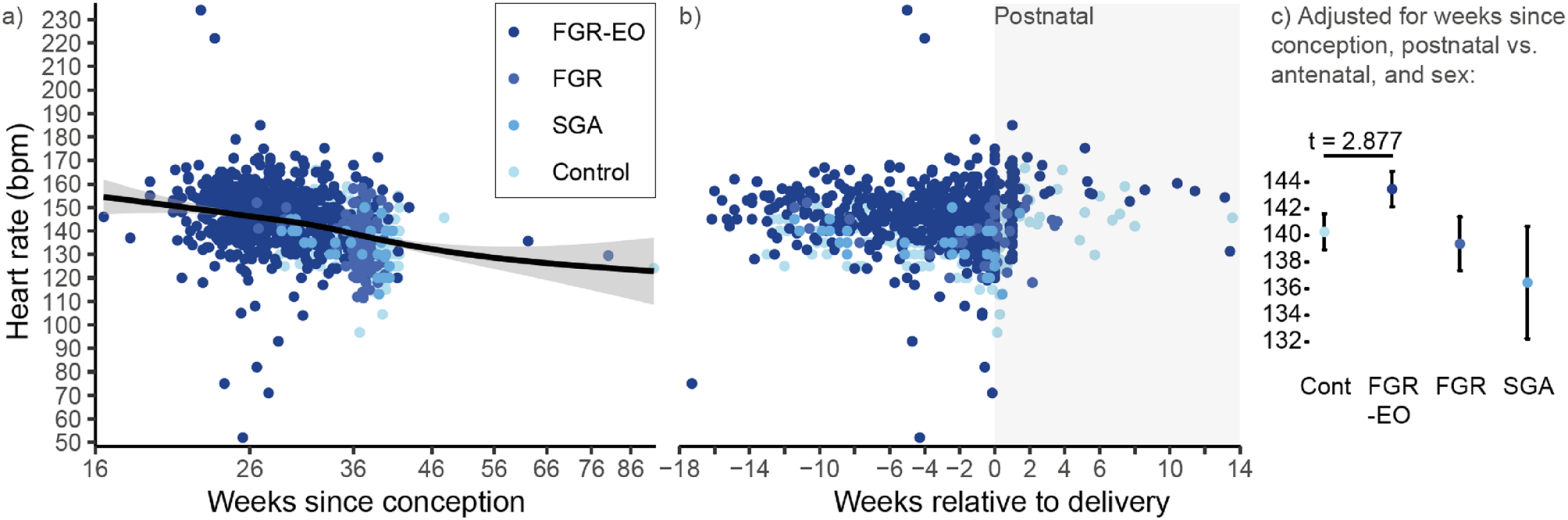
After adjusting for weeks since conception **(a)** and postnatal vs. antenatal **(b)** at measure, FGR-EO subjects had a higher heart rate at rest than controls **(c).** In a), grey shading indicates standard error of the mean; note that the x axis is logarithmic. In b), for illustrative purposes, the effect of postnatal vs. antenatal status is visualised by plotting those 976/1242 measures for which weeks since conception at delivery was available, as this makes apparent the inflection point at transition from antenatal to postnatal. In c), the estimated adjusted means and 95% confidence intervals are displayed (control: 139–142, FGR-EO: 142–145, FGR: 137–141, SGA: 132–141 bpm). Note that only controls and FGR-EO have non-overlapping confidence intervals, while the wider intervals for FGR and SGA - overlapping with the control confidence interval - indicate greater uncertainty about the position of the mean in those groups.

Across subjects, heart rate declined by −0.5 beats per minute/week since conception at measure (t = −5.645; Fig. 1a). Postnatal heart rates were higher than antenatal heart rates (mean 147.1 vs. 140.8, t = 6.870; Fig. 1b). FGR-EO (but not FGR or SGA) subjects had a higher heart rate than controls (mean 143.5 vs. 140.3, t = 2.877 (1.7% of controls and 1.4% of FGR-EO subjects were multiple gestation); Fig. 1c). This did not interact with weeks since conception at measure (AIC worsened from 9541.8 to 9545.1) or postnatal vs. antenatal status (AIC negligible change to 9541.3). Taken together, the higher heart rate in the FGR-EO group relative to controls persisted through the developmental period examined with no evidence of this difference closing or widening.

Figures 1a-b show that some measures were physiological outliers, so we went on to fit the data using a robust estimation method that down-weighs outliers^77^, to ensure our results were accurate. The coefficients of this model were approximately the same, but had smaller standard errors which resulted in larger t values (decline in heart rate with weeks since conception at measure, t = −7.17; higher postnatal heart rates, t = 8.40; higher heart rate in FGR-EO than controls, t = 3.47).

We went on to examine the effect of stratifying subjects differently. Concordant with our finding that the smallest (FGR-EO) subjects had higher heart rates, for every 100 fewer grams of body weight at time of measure, heart rate was 0.2 beats per minute higher (t = 2.199, *n* = 430 subjects, 876 measures). On the other hand, the coarser classification metric of < 10th birth weight centile (yes/no) was not associated with heart rate (448 subjects, 980 measures). Finally, we confirmed that postnatal heart rate was higher with fewer weeks since conception at birth (across groups) in line with previous work^78,79^, and not associated with its method of calculation (Supplementary).

#### Postnatally, FGR-EO subjects exhibited a blunted capacity to increase heart rate to a physiological challenge

Next, we assessed whether heart rate *reactivity* differed in FGR infants relative to controls, to a physiological challenge (clinically necessary nociceptive procedure (heel lance)). This analysis included 46 subjects born between 26 and 38 weeks since conception, with 47 measures between 30 and 39 weeks since conception (21 control (median 32 weeks + 1 day at birth (32 + 1) and 35 + 0 at measure), 16 FGR-EO (median 31 + 1 at birth and 33 + 4 at measure), 8 FGR (median 36 + 1 at birth and 37 + 3 at measure; 1 repeated measure), 2 SGA (both were 38 weeks at birth and 39 weeks at measure).

**Fig. 2:**
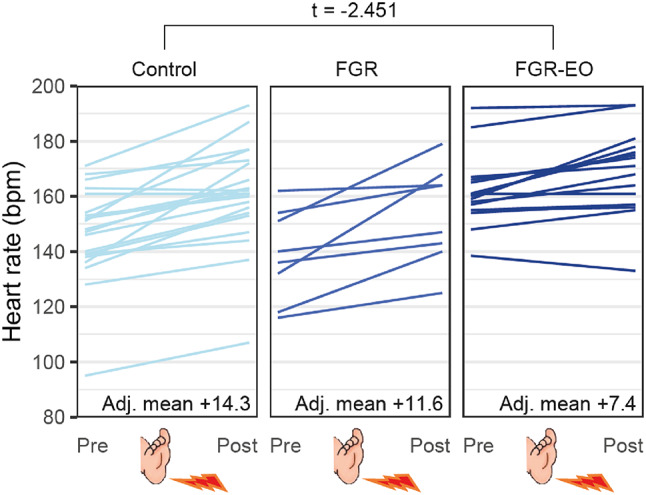
FGR-EO (but not FGR) subjects exhibited a blunted capacity to increase heart rate to a physiological challenge (clinically necessary heel lance) relative to controls. In each panel, the ‘Adj. mean’ reported at the bottom is the estimated mean change adjusted for weeks since conception at birth, weeks since conception at measure, and sex.

Across subjects, heart rate change increased by 1.6 beats per minute/week since conception at measure (t = 2.280) but was not associated with weeks since conception at birth. FGR-EO infants exhibited a smaller heart rate increase relative to controls (mean 7.4 vs. 14.3 beats per minute, t = −2.451 (13% of FGR-EO subjects and 19% of controls were multiple gestation); Fig. 2). While FGR infants did not differ from controls, their heart rate increase was also slightly smaller (mean 11.6 vs. 14.3 beats per minute; Fig. 2) (too few SGA subjects to compare). There was no interaction between weeks since conception at measure and group (AIC worsened from 346.9 to 351.3).

Finally, we examined the effect of stratifying subjects differently. For body weight at measure, only 37 of the original 47 data points had that information available: in this smaller sample, this was not a significant predictor. Infants < 10th birth weight centile exhibited a smaller heart rate increase than infants ≥ 10th centile (mean 7.5 vs. 14.3 beats per minute, t = −2.589).

### FGR-EO infants had smaller relative white matter volume

**Fig. 3:**
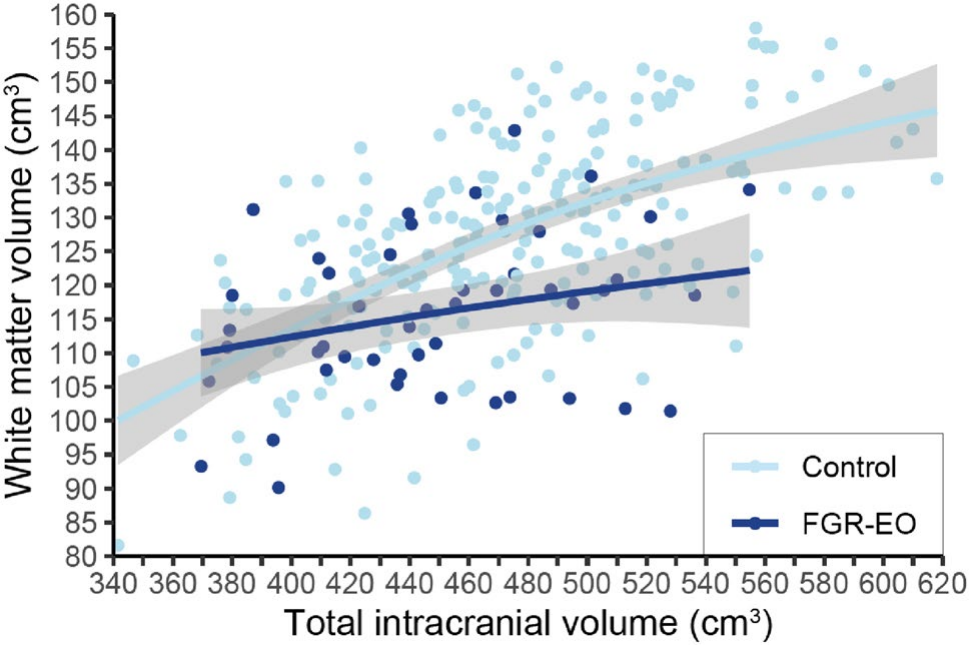
FGR-EO infants had smaller white matter volume, even after accounting for total intracranial volume. Grey shading indicates standard error of the mean.

Structural MRI data were analysed from 270 subjects born between 23 and 32 weeks since conception and scanned between 38 and 45 weeks since conception at a single MRI department^49^ (47 FGR-EO (median 30 + 1 at birth and 42 + 3 at MRI); 223 controls (median 30 + 2 at birth and 43 + 0 at MRI); no repeated measures). White matter volume was 1.0 cm^3^ higher/week since conception at birth (t = 3.194), 0.2 cm^3^ higher/1 cm^3^ total intracranial volume (t = 12.255), and 3.0 cm^3^ lower/week since conception at MRI (t = −6.218; as white matter volume was normalised by total intracranial volume in this model, this reflects its *relative* developmental decline: see Fig. 13 in^80^). FGR-EO subjects had mean 7 cm^3^ lower white matter volume than controls (mean 119.4 vs. 126.5 cm^3^, t = −4.065). There was no interaction between group and weeks since conception at delivery (AIC worsened from 2029.2 to 2031.2), weeks since conception at MRI (AIC worsened to 2029.6), or total intracranial volume (AIC negligible change to 2026.5). In sum, FGR-EO subjects had less white matter than would be expected, even for their small head size (Fig. 3). This was reproduced in a sensitivity analysis using singletons alone (Supplementary).

Next, we examined the effect of stratifying subjects differently. Using birth weight as an alternative independent variable (as body weight at MRI was not available for any data points), for every 100 fewer grams of birth weight, white matter volume was 0.768 cm^3^ lower (t = −2.782). Infants < 10th birth weight centile had 6 cm^3^ lower white matter volume than infants ≥ 10th centile (mean 119.8 vs. 126.1 cm^3^, t = −3.181).

### Body weight across development

We went on to model body weight longitudinally, from the antenatal period (estimated fetal weight) through the postnatal period (actual weight). This was the largest analysis, comprising total 1660 subjects: every subject (Table 1) was included except for the 262 from the antenatal IEEE dataset (they did not have any weight data and were only used in the heart rate at rest analysis). The 1660 subjects had total 7971 measures (antenatal: postnatal 2849:5122 (36%:64%)) (FGR-EO: 239 subjects, 1567 measures (47% antenatal); FGR: 104 subjects, 610 measures (40% antenatal); SGA: 311 subjects, 1237 measures (5.1% antenatal); controls: 1006 subjects, 4557 measures (40% antenatal)). Body weight was slightly more similar within sites (ICC: 0.02), and therefore both subject and site were included as random effects. In the next section we report the fixed effects, and in the subsequent section the random effects.

#### FGR subjects continued to have lower body weight to six years of age

**Fig. 4:**
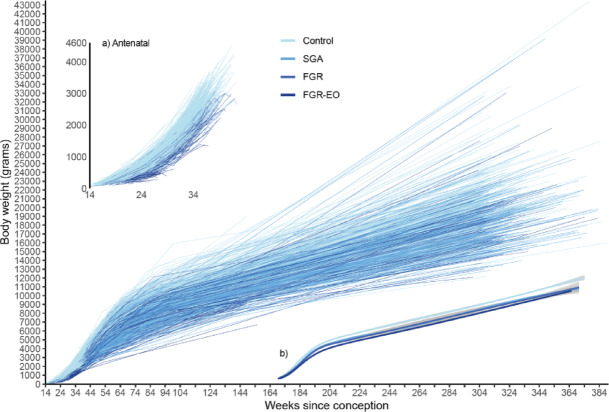
FGR subjects continued to have lower body weight to six years. Main: Subject-level body weight trajectories, pooled from the antenatal period (estimated fetal weight) through the postnatal period, colour-coded by group. (a) Plotting the antenatal measures alone confirmed the small fetal size expected in the non-control groups, most marked in FGR-EO. (b) Group-level mean body weight trajectories, across the same developmental period as the main figure; grey shading indicates standard error of the mean, note that the SGA and FGR mean trajectories are overlapping, with the wider standard error belonging to the SGA group.

Between 14 weeks since conception and six years, three developmental phases of weight gain were apparent: relatively modest weight gain to approximately 25 weeks since conception, a subsequent acceleration to 70 weeks since conception, and then a deceleration from this point (approximately 6 months old) (Fig. 4). To model this, we created a piecewise regression model (Fig. S1), including an interaction term with group (AIC improved from 134,936.0 to 134,634.3). Overall (across groups), the coefficients of the three pieces were 738, 8674, and 23,482 g. After removing the weight gained during the previous piece when applicable, calculated slopes were: 67 g/week between 14 and 25 weeks since conception, 176 g/week between 25 and 70 weeks since conception ((8674 − 738)/45 weeks), and 46 g/week between 70 and 390 weeks since conception ((23,482–8674)/320 weeks). Put otherwise, there was a change in slope of + 109 g/week at 25 weeks since conception and − 130 g/week at 70 weeks since conception.

To examine the effect of group on weight, we reviewed the interaction terms for the second and third pieces of the regression (we disregarded the first piece to avoid circularity, as group membership had been assigned based on fetal growth). Between 25 and 70 weeks since conception, FGR-EO and FGR subjects were mean 2194 and 1186 g lighter than controls (t = −7.008, −3.809; SGA n.s.: mean 1026 g lighter but large s.e.). Between 70 and 390 weeks since conception, FGR-EO and FGR subjects were mean 2508 and 1759 g lighter than controls (t = −5.816, −4.886; SGA n.s.: mean 1691 g lighter but large s.e.). Next, we tested whether - accounting for their lower weight at the onset of each piece - FGR-EO, FGR and SGA subjects had lower weight *gain* than controls. Between 25 and 70 weeks since conception, FGR-EO and FGR subjects gained mean 33 and 14 fewer grams/week than controls (t = −12.908, −3.668; SGA n.s.). Between 70 and 390 weeks since conception, FGR and SGA subjects gained mean 2 fewer grams/week than controls (t = −2.327 and − 4.251; FGR-EO n.s.: mean 1 fewer gram/week but large s.e.). Finally, we examined whether group affected the change in slope at 25 and 70 weeks since conception. Relative to controls, the change in slope at 70 weeks since conception was + 32 and + 12 g/week in FGR-EO and FGR, implying slightly potentiated growth momentum at the point when weight gain decelerates (t = 10.002 and 2.839; SGA n.s.) (25 weeks: all n.s.). These results were reproduced in sensitivity analyses using singletons alone (Supplementary).

In the model of integrated data described above, there was no effect of postnatal vs. antenatal status. Last, we constructed the same model for the 5122/7971 measures that were postnatal. As just 12 postnatal measures were available before 25 weeks, we removed the knot at 25 weeks from the piecewise model. Weeks since conception at delivery was not associated with body weight (AIC negligible change from 88,565.0 to 88,562.1). In sum, FGR-EO and FGR subjects had lower body weight than controls for the full developmental period examined (Fig. 4b), with no evidence that this was explained by differences in weeks since conception at birth.

#### The effect of FGR on body weight across development was robust to site effects

We reviewed the random effects of site and subject on the antenatal-postnatal body weight model intercept. A more negative random effect indicates that the site or subject’s individual regression line was lower than predicted by the model, while a more positive random effect indicates that the site or subject’s individual regression line was higher than predicted by the model.

The random effect of site ranged from − 220 to + 267 g. The FEMINA2, Norway, and Alabama sites were associated with positive random effects, while the Lund, Barcelona, UCLH, Hamburg, and ePrime sites were associated with negative random effects. We re-calculated the fixed effects coefficients with each site deleted in turn. All the statistically significant effects reported above persisted with the deletion of any of the sites. For example, between 25 and 70 weeks, FGR-EO and FGR subjects had body weights that were 1626–2286 and 990–1252 g lower than controls, depending on which site was deleted.

Body weights were more clustered within subject than within site (ICC 0.15 vs. 0.02), with subject random effect 25th and 75th quartiles of −178 and + 139 g (Fig. 4 main visualises this inter-subject variance). In the next section, we tested whether this inter-subject variance in body weight was associated with variance in neurodevelopmental outcomes. To do this, we entered the random effect of each subject as an explanatory variable for outcome.

### Neurodevelopmental outcomes

A motor score was available for 1030 subjects, born between 23 and 43 weeks since conception (of these: 654 (63%) controls 24–43 weeks; 196 (19%) SGA 29–43 weeks, 61 (6%) FGR 33–42 weeks, 119 (12%) FGR-EO 23–41 weeks). A cognitive score was available for 1038 subjects (almost the same demographics as for motor score). We did not analyse language because this score was available in less than a third of instances (309 subjects), and only in controls and FGR-EO (just one/309 FGR).

#### Prematurity, FGR, and poorer individual growth, were associated with lower motor score

Motor score was more similar within sites than between sites (ICC: 0.16) and therefore site was included as a random effect. Here we report the fixed effects, and in the next section we describe the site effect. Motor score was 1 point lower/1 fewer week since conception at birth (t = −7.640). It was 5, 5, and 3 points lower in FGR-EO, FGR, and SGA relative to controls (mean 105, 105, and 107 relative to 110, t = −3.310, −2.893, −2.684). Motor score was also lower with more negative growth random effect: 0.4 points lower/−100 g (t = −3.402). There was no interaction between growth random effect and weeks since conception at birth or group (AIC negligible change from 8199.8 to 8198.2 or 8199.2 respectively). Entering an interaction term between weeks since conception at birth and group reduced AIC but produced illogical coefficients due to collinearity between the interaction term and the main effect of group (GVIFs 12.8 vs. 1.00–1.18 for the other predictors). A motor score more than one standard deviation below the reported population mean is commonly interpreted as an adverse outcome, which was the case for 53/1030 subjects (5.1%). Therefore we re-ran the model using motor score < 85 (yes/no) as the dependent variable. Adverse motor outcome was 1.22 times more likely/1 fewer week since conception at birth (z = 3.912) and 4.88 times more likely in FGR-EO (but not FGR or SGA) relative to controls (z = 4.890), but not associated with growth random effect. Children with a motor score ≥ 85 were median 38 weeks since conception at birth, and 9.4% were FGR-EO. Children with a motor score < 85 were median 30 weeks since conception at birth, and 51% were FGR-EO.

Next, we returned to the primary model (motor outcome as a continuous variable) and examined the effect of stratifying subjects differently. Using birth weight as an alternative independent variable (as body weight at neurodevelopmental assessment was not consistently available), for every 100 fewer grams of birth weight, motor score was 0.3 points lower (t = −2.775). Infants < 10th birth weight centile had lower motor scores (mean 106 vs. 109, t = −4.185) than infants ≥ 10th centile. The association with growth random effect persisted in both models (0.3 and 0.4 points lower/−100 g respectively, t = −2.553 and − 3.355).

#### The effects of FGR and poorer individual growth - but not SGA - on motor score were robust to site effects

The random effect of site on motor score ranged from − 6 to + 8 points. The Alabama, Norway, and Lund sites were associated with positive random effects, while the Barcelona, UCLH, Hamburg, and ePrime sites were associated with negative random effects. We re-calculated the fixed effects coefficients with each site deleted in turn. The effect of weeks since conception at birth, FGR-EO and FGR, and growth persisted with the deletion of any of the sites (weeks since conception at birth: always 1 point lower/1 fewer week; FGR-EO and FGR: 4–6 and 2–5 points lower than controls respectively; growth: 0.4–1.0 points lower/−100 g). However, the deletion of the Alabama site reversed the direction of the SGA fixed effect, which was therefore considered unreliable (note that 91% of the SGA motor scores derived from that site).

#### Prematurity, FGR-EO, and poorer individual growth in FGR-EO subjects, were associated with lower cognitive score

Cognitive score was more similar within sites than between sites (ICC: 0.44) and therefore site was included as a random effect. Here we report the fixed effects, and in the next section we describe the site effect. We included an interaction term between group and growth random effect (AIC improved from 7658.6 to 7652.9). Cognitive score was 0.9 points lower/1 fewer week since conception at birth (t = −7.953). It was 4 points lower in FGR-EO (but not FGR or SGA) relative to controls (mean 106 vs. 110, t = −3.476). Cognitive score was not overall associated with growth random effect, but this interacted with FGR-EO (extra 1.5 points lower/−100 g in FGR-EO relative to controls, t = −3.408). There was no interaction between growth random effect and weeks since conception at birth (AIC worsened to 7653.6). As for motor score, entering an interaction term between group and weeks since conception at birth produced collinearity between the interaction term and the main effect of group (GVIFs 13.2 vs. 1.01–1.33 for the other predictors). Fifty-seven/1038 subjects (5.5%) had a cognitive score < 85. A binomial model using cognitive score < 85 (yes/no) as the dependent variable struggled to converge, so we reduced its complexity by removing the interaction term between group and growth random effect. In this model, adverse cognitive outcome was 1.39 times more likely/1 fewer week since conception at birth (z = 5.584; children ≥ 85 or < 85 were median 39 and 28 weeks since conception at birth respectively). Adverse cognitive outcome was not associated with group (FGR-EO n.s. but borderline z = 1.955; children ≥ 85 or < 85 were 10% and 37% FGR-EO respectively) or growth random effect. Next, we returned to the primary model (cognitive outcome as a continuous variable) and examined the effect of stratifying subjects differently. Neither birth weight nor < 10th birth weight centile (yes/no) predicted cognitive score.

#### The effects of FGR-EO and poorer individual growth in FGR-EO subjects on cognitive score were robust to site effects

The random effect of site on cognitive score ranged from − 12 to + 14 points (same sites associated with positive or negative random effects as for motor score). We re-calculated the fixed effects coefficients with each site deleted in turn. The effect of weeks since conception at birth, FGR-EO, and the FGR-EO vs. growth interaction persisted with the deletion of any of the sites (weeks since conception at birth: 0.9–1.0 point lower/1 fewer week; FGR-EO: 1–5 points lower than controls (1 lower with the deletion of ePrime, 4 to 5 points lower with the deletion of any other site); interaction: 0.6–1.7 points lower/−100 g.

**Fig. 5:**
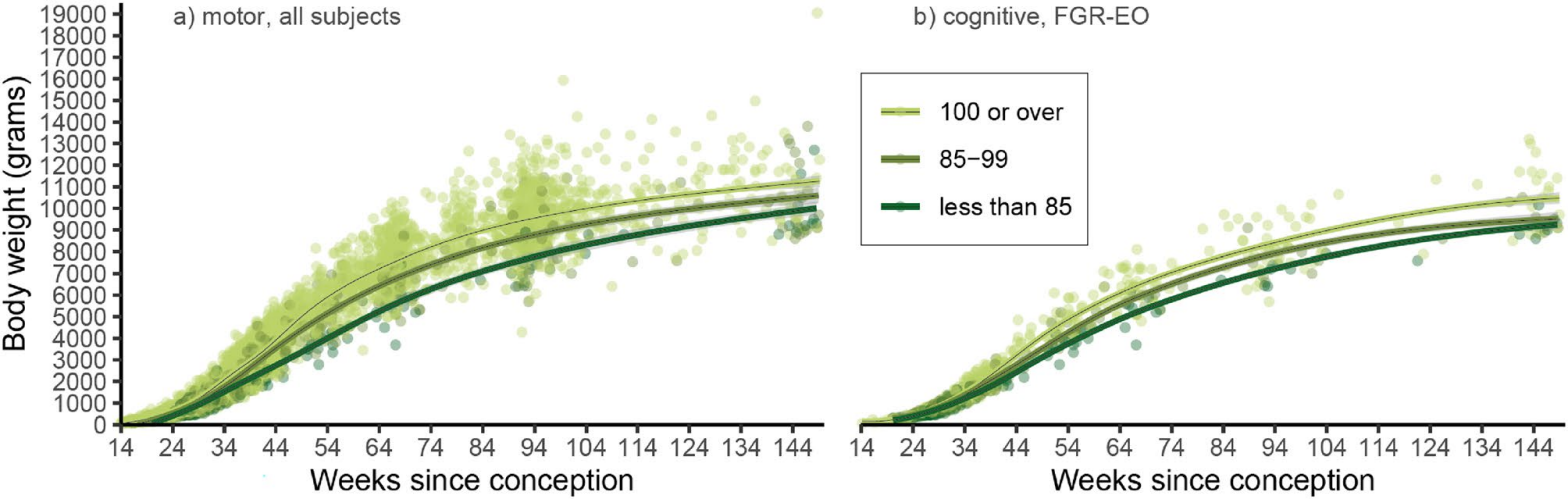
Poorer weight gain was associated with lower motor score across groups **(a)**, and lower cognitive score in FGR-EO subjects specifically **(b).** To visualise overlapping values, body weight data points are semi-transparent. For illustrative purposes, the association between weight gain and neurodevelopmental outcome is visualised by plotting mean body weight trajectories, colour-coded by three outcome groups (motor all subjects: ≥100 = 80%, 85–99 = 15%, < 85 = 5%; cognitive FGR-EO: ≥100 = 50%, 85–99 = 32%, < 85 = 18%). Note that the x axis stops at 2 years (150 weeks since conception) for consistency with the latest point that neurodevelopmental outcomes were assessed. Grey shading indicates standard error of the mean but is minimal when the data are dense and therefore difficult to appreciate.

To summarise the neurodevelopmental results overall: FGR-EO and FGR, and poorer individual growth across groups (no interaction with group), was associated with lower motor score (Fig. 5a). FGR-EO specifically, and poorer individual growth in FGR-EO subjects alone, was associated with lower cognitive score (Fig. 5b). This was reproduced in sensitivity analyses using singletons alone (Supplementary). When using < 85 as a cut-off, FGR-EO singly was predictive, implying that the effects of FGR and poorer individual growth were largely associated with lower neurodevelopmental scores within the normative range. To visualise this, we plotted body weight across development for children at or above the reported population mean, below the mean but within 1 standard deviation, and more than 1 standard deviation below the mean (Fig. 5).

## Discussion

In this study examining how metabolic differences develop, or manifest in growth trajectories, in fetuses and children from FGR, SGA, and normal pregnancies, our first finding is that FGR-EO subjects continue to exhibit higher whole-body metabolic rate for several weeks postnatally^24,81,82^. Extrapolating from their perinatal heart rate being approximately 3.2 beats per minute faster, their heart will beat around 4600 times more per day across this period. Or, put another way, their heart beats at the rate of a subject 6 weeks younger. This effect of FGR-EO was independent of the effect of intra- to extra-uterine transition, which also increased heart rate. Previous work has shown that metabolic rate rapidly increases in the few days after birth^13–15^. Here, our integration of fetal-neonatal data allowed us to capture this phenomenon across the point of birth, reiterating the value of modelling intra- to extra-uterine continuity.

FGR-EO subjects’ higher heart rate at rest may explain the origin of their dampened autonomic heart rate control, which is unmasked by a physiological challenge^8,25^. There is concordant evidence in adults that when the energy budget is constrained (through elite-level sports training), heart rate responses to stressors are dampened^83^. This is inferred to minimise the energy expenditure associated with each extra beat per minute^84^. Although previous studies have suggested that FGR infants do not respond to physiological challenges with the usual increase in heart rate, those stimuli lacked ecological validity (breathing CO_2_, tilt table)^81,85^. Here we show that FGR-EO infants demonstrate lesser heart rate reactivity to a naturalistic challenge that a hospitalised infant will regularly face. This complements our previous work showing that another group of vulnerable infants - those with higher physiological stress levels - also appear hypo-responsive to this same challenge^86^. These data highlight the importance of accounting for inter-individual differences in physiological baseline, as infants maintain allostasis in a changing sensory environment.

As well as exhibiting evidence of higher whole-body metabolic rate (indexed by heart rate at rest), our results imply that FGR-EO subjects counteract this by minimising a major energy expenditure - brain metabolic rate - via reducing synapse number (indexed by white matter volume)^31–33^. Older FGR infants, at 1 year of age, also show reduced white matter volume, although with regional specificity^87^. In neonatal mammalian models, the development and maintenance of synapses requires post-synaptic activity^88,89^. Therefore, in human subjects with FGR, lower synapse number could be mediated by attenuated post-synaptic activity. In line with this hypothesis, electroencephalography - which indexes post-synaptic activity - can differ in FGR infants, although with considerable discrepancies across studies^90^.

While FGR subjects adopt strategies to reduce energy expenditure^26–28^, faltering growth indicates a residual shortfall. Our results imply that their abnormal fetal growth reprogrammes growth into childhood. We show that FGR subjects have potentiated growth momentum at the point when weight gain decelerates at approximately 6 months of age, which is likely to be adaptive. Nevertheless, they are overall ill-resourced for postnatal growth and stay small until at least six years, failing to catch up with their peers even though they have left the compromised uterine environment. By stratifying the cohort into four groups, our growth curves visualise the increasing impact on growth to six years of being SGA, FGR, or FGR-EO, relative to controls. We then show how poorer *individual* growth, beyond that explained by group, is independently associated with lower motor score across subjects, and lower cognitive score in FGR-EO specifically^91^. Therefore, universally, including in controls, poorer growth is associated with lower motor score, meaning that this effect does not require in utero growth restriction first^92^. However, in FGR subjects, especially FGR-EO, there appears to be an additive effect of two disadvantages: FGR group status (reflecting the presence of in utero growth restriction), and poorer *individual* growth beyond that explained by group. Consequently, growth trajectories encode information about how FGR is transmitted into suboptimal neurodevelopment.

A strength of this work is that we took account of its multi-site nature to identify to what extent findings were robust to site influences. Neurodevelopmental scores were the only dependent variable notably clustered within site, with site ICC values in line with other studies (e.g. Table 3 in^93^). As social advantage is associated with enrolment in research and retention at follow-up^94,95^, which likely explains higher mean neurodevelopmental scores here than the reported population mean, this clustering may arise from inter-site differences in study protocols exacerbating or ameliorating these trends. Furthermore, previous work has shown effects on neurodevelopmental score of hospital of birth and examiner of up to 13 and 7 points respectively^96^. For motor score, all effects - except that of being SGA - were robust to these site influences. The unreliability of the SGA finding likely reflects a negligible effect of being SGA when there is no evidence of FGR, such that the error around the correct estimate of effect crosses zero. Indeed, although being SGA has been associated with minor neurodevelopmental difficulties, this effect is not conserved when children are compared against matched siblings^97^, implying that it is not SGA that confers risk, but associated family-level variables. For cognitive score, all effects were robust to site influences, although we noticed an out-sized impact of the ePrime dataset, such that the effect of being FGR-EO was smaller without its inclusion. A possible explanation is that it was the only dataset in which 100% of recruitment was postnatal and involved enrolment into brain MR imaging. Feasibly, parents may already have received postnatal information that resulted in concerns about their infant’s cognitive development, increasing their chance of saying Yes to this MRI research. In line with this possibility, in other neonatal neurology research when parents were approached after having received information about their child’s condition, parental consent was higher in the case of more serious illness^95^.

How FGR is defined, and dissociated from SGA, has varied across the literature^52^. Using < 10th birth weight centile as a proxy for FGR has been valuable in global health research, as this information is available for around four fifths of countries^46^. However, the SGA definition encompasses constitutionally small but healthy subjects, and excludes growth-restricted subjects > 10th centile. For example, the SGA definition would include a subject on the 9th centile even though that is their genetic growth potential, but exclude a subject on the 13th centile who had the genetic growth potential to be on the 75th centile and has experienced placental insufficiency. The recent Delphi consensus definition recommends that Doppler ultrasound information is used to support the diagnosis of FGR^52^, but this was unavailable for many of the data points here so we sometimes dissociated FGR from SGA using evidence of antenatal adversity (Methods; Table S1). When we assessed how results varied if infants were instead binarised into < 10th birth weight centile (yes/no), differences were usually lost (heart rate at rest^79^; cognitive outcome) or had weaker effect sizes (MRI; motor outcome). This indicates the value of our classification method that captured the biological gradient of risk. Consistent with this, our second sensitivity analysis - placing subjects onto a continuous scale according to body weight - more often showed similar results (e.g. heart rate at rest) so long as sample sizes were comparable. We also assessed the validity of our classification method by reviewing the effect of excluding subgroups, chiefly multiple gestation subjects. We did this because we categorised these subjects using standard (singleton) growth charts, which has higher sensitivity but lower specificity to detect clinically significant small size relative to twin growth charts^54,98^. If this resulted in misclassification, we would expect the inclusion of multiples to ‘dilute’ any effect size between SGA or FGR vs. controls. If so, a sensitivity analysis excluding multiples would unmask additional difference in SGA or FGR vs. controls. However, sub-analyses that excluded multiples were comparable to the full analyses (Supplementary).

Aside from heterogeneity across sites, and the limitation of not always having access to Doppler information to aid classification, discussed above, this work has other weaknesses. While the majority of analyses benefited from large sample sizes, other data samples were modest, including the heart rate reactivity part. Further, we would have liked to model subject-level random effects on slope (e.g. growth rate) in addition to intercept, but these more complex models would not converge, which is a common issue^65^. Nevertheless, individual differences in growth across healthy children are largely explained by different intercepts, rather than different slopes^99^. Beyond this, we modelled growth using body weight rather than height or head circumference, because weight alone is formally related to metabolic rate^8,100^ and was also more widely available across our dataset. However, those other measures of growth are also relevant^101^. Also, we were unable to account for nutritional practices, although there is little evidence that they notably impact weight gain or neurodevelopment when other factors are adjusted for (Supplementary Discussion). Furthermore, while we report socio-economic and ethnicity information when available (Table S1), this study was not set up to address the influence of those factors although they are likely to have contributed^47^. Finally, we only studied neurodevelopment within motor and cognitive domains, but FGR can also affect language and wider social communication^49,102^.

In conclusion, this is the largest work - to our knowledge - which includes longitudinal fetal-postnatal courses associated to FGR. Its comprehensive study of different developmental domains (cardiac, autonomic, neurological, physical, behavioural) shows the complex effects of FGR, which are uniquely evident when integrated together within a unified metabolic framework. We provide converging and multidimensional evidence of persistent metabolic and neurodevelopmental differences after FGR. Together, our results represent FGR as an evolving neurological compromise which reaches far into the postnatal period, and in which metabolic and autonomic adaptations to the initial insult may further harm development, beyond the initial antenatal insult. Growth trajectories encode unique information about how FGR is transmitted into suboptimal neurodevelopmental outcomes, and could identify intervention opportunities in this vulnerable population.

## Electronic Supplementary Material

Below is the link to the electronic supplementary material.

Supplementary Material 1

## Data Availability

Please direct enquiries to the corresponding author.
