# Supplementary material for: Metabolic expenditure, neurodevelopment, and weight gain into early childhood after fetal growth restriction
Source: Sci Rep. 2026 Jun 15;16:17841. doi: 10.1038/s41598-026-53713-y (PMC13270132; doi:10.1038/s41598-026-53713-y)
Supplement: Supplementary file 1 — Supplementary Material 1 [file 41598_2026_53713_MOESM1_ESM.docx]

**Metabolic expenditure, neurodevelopment, and weight gain into early childhood after fetal growth restriction**

Cigdem Gelegen^a§^, Beatrice Copley^b§^, Neelum Mistry^c§^, Chiara Sacchi^d^, Chiara Nosarti^ef^, Lorenzo Fabrizi^c^, Anna L David^a^, Kimberley Whitehead^abc*^

^a^ Elizabeth Garrett Anderson Institute for Women’s Health, University College London, London, WC1E 6AU, UK

^b^ Research Division of Digital Health and Applied Technology Assessment (DHATA), King’s College London, London, SE1 8WA, UK

^c^ Department of Neuroscience, Physiology and Pharmacology, University College London, London, WC1E 6BT, UK

^d^ Department of Developmental Psychology and Socialisation, University of Padua, Padua, 35122, Italy

^e^ Department of Early Life Imaging, School of Biomedical Engineering and Imaging Sciences, King’s College London, London, SE1 7EH, UK

^f^ Department of Child & Adolescent Psychiatry, King’s College London, London, SE1 7EH, UK

^§^Contributed equally

**Contents – and their page numbers - within Supplementary material**

| **Supplementary content** | **Page** |
| --- | --- |
| Table S1: Details about each data source | 2 |
| Table S2: Which measures were available for each data source | 3 |
| Supplementary to Methods/*Subjects*: When FGR was dissociated from SGA without Doppler measurements, FGR classification was associated with markers of placental insufficiency | 4 |
| Supplementary to Heart rate: Postnatally, lower weeks since conception at birth predicted higher heart rate | 5 |
| Supplementary to FGR-EO infants had smaller relative white matter volume: sensitivity analysis re. influence of multiple pregnancies | 6 |
| Supplementary to Body weight across development: visualisation of model  Fig. S1: Piecewise regression model of the effect of weeks since conception on body weight. | 7 |
| Supplementary to Body weight across development: sensitivity analysis re. influence of multiple pregnancies | 8 |
| Supplementary to Neurodevelopmental outcomes: sensitivity analysis re. influence of multiple pregnancies | 9 |
| Supplementary to Discussion: There is no evidence from existing literature that nutritional practices notably impact growth or neurodevelopment when other factors are adjusted for | 10 |
| Bibliography | 11 |

**Table S1: Details about each data source**

| Dataset | Site | Maternal ethnicity ^a^ | Maternal paid employment ^a^ | Rec | Subs | Included multiple pregnancies | FGR distinguished from SGA using Doppler measurements |
| --- | --- | --- | --- | --- | --- | --- | --- |
| neuro | UCLH, London, England | ND ^b^ | ND ^b^ | P | FGR-EO, FGR, SGA, Controls | Y | Y |
| EVERREST ^c^ | UCLH, London, England | 51% White  23% Black  19% Asian  7% Other | Y 77.2%  N 13.9%  U 8.9% | A | FGR-EO | N | NA |
| EVERREST | Hamburg,  Germany | 82.8% White  3.45% Black  3.45% Asian  10.3% Other | Y 82.8%  N 10.3%  U 6.9% | A | FGR-EO | N | NA |
| EVERREST | Lund,  Sweden |  |  | A | FGR-EO | N | NA |
| EVERREST | Barcelona,  Spain |  |  | A | FGR-EO | N | NA |
| ePrime | 14 hospitals, London,  England | 56% White  18% Black  24% Asian  2% Other ^d^ | ND ^g^ | P | FGR-EO, Controls | Y | NA |
| FEMINA2 | Manchester, England | 65% White 12% Black 17% Asian  6% Other ^e^ | ND | A | FGR-EO, FGR, SGA, Controls | N | Y |
| IEEE | Naples,  Italy | ND | ND | A | FGR, Controls | N | NA |
| Norway-Alabama | Trondheim and Bergen, Norway &  Uppsala, Sweden | 100% White | Y 74.8%  N 16.7%  U 8.5% ^h^ | A,P | FGR-EO, FGR, SGA, Controls | N | N |
| Norway-Alabama | Alabama,  USA | 25% White 75% Black ^f^ | Y 40.1%  N 50.6%  U 9.3% ^i^ | A,P | FGR-EO, FGR, SGA, Controls | Y | N |

neuro = neurophysiological (see main manuscript: Methods/*Data*). UCLH = University College London Hospitals. Rec = Location of recruitment. A = Antenatal. P = Postnatal. Subs = Subgroups.

ND = Not done, i.e. not available. NA = Not applicable. Y = Yes. N = No. U = Unknown.

^a^ In the cells of the table, we provide these data for all included subjects from that dataset (irrespective of group); if statistically significant association with group, this is detailed in footnotes. ‘Other’ ethnicity = mixed ethnicity or other ethnicity not stated.

^b^ Although not available, it would be reasonable to assume that proportions might be broadly comparable to the EVERREST cohort recruited at the same site (row below).

^c^ As the majority of EVERREST subjects derived from UCLH (104/133), we provide demographic data for UCLH, and then the other sites pooled.

^d^ Ethnicity-group association. Chi-squared: post-hoc Bonferroni-adjusted pairwise tests indicated that only White ethnicity differed between groups (p = 0.04; 60% in controls and 37% in FGR-EO). As White mothers have a lower chance of an FGR pregnancy in England [1] (the location of the ePrime cohort) and other multi-ethnic high-income societies [2], the ePrime cohort therefore reflects the demographic distribution of the condition.

^e^ Ethnicity was redacted at the single-subject level so an ethnicity-group comparison was not possible, but ethnicity proportions across the overall cohort could be ascertained from Table S2 in [3].

^f^ No ethnicity-group association. Chi squared p = 0.28.

^g^ Maternal employment data not available, but index of multiple deprivation quintiles (missing n = 5) was not associated with group, as previously reported [4]. Chi-squared p = 0.79.

^h^ No paid employment-group association once a smaller percentage of missing data in the control group was accounted for. Chi-squared: post-hoc Bonferroni-adjusted pairwise tests indicated that only missing data differed between groups (p = 0.02; 3% in controls vs. 16-33% in other groups).

^i^ Paid employment-group association. Chi-squared: post-hoc Bonferroni-adjusted pairwise tests indicated that only controls differed from the other groups (p = 0.03; 49% in paid employment vs. 33-42% in other groups). As mothers with higher socio-economic advantage have a lower chance of an FGR pregnancy [2], the Alabama cohort therefore reflects the demographic distribution of the condition.

**Table S2: Which measures were available for each data source**

| Dataset | HR | HR react | MR | Body weight | Bayley  scores ^a^ |
| --- | --- | --- | --- | --- | --- |
| neuro | A: if CTG clinically indicated  P: during neurophysiological research study (pulse oximetry or ECG-derived) | Y | N | A always: ≥15, 28, 32, 36 weeks, ≤1/week  B  P: +/- 2 days of neurophysiological research study, hospital discharge, 3 mths, 6 mths, 1 yr, 2 yrs, other appointments ≤5 yrs | 1 or 2 yrs |
| EVERREST | A: if CTG clinically indicated  P: day of birth (not *at* birth), 7 days  (pulse oximetry-derived as logged in daily medical notes) | N | N | A always: ≥16 weeks, ≤1/week  B  P: 7 days, hospital discharge, 3 mths, 6 mths, 1 yr, 2 yrs | 1 or 2 yrs |
| ePrime | N | N | Y | B  P: hospital discharge | 2 yrs |
| FEMINA | A always: n = 1 ≥28 weeks | N | N | A always: n = 1 ≥28 weeks  B | N |
| IEEE | A always: n = 1 ≥36 weeks | N | N | N | N |
| Norway-Alabama | N | N | N | A in 84%: 14-18, 22-26, 30-34, 36-38 weeks  B  P: 6 weeks, 3 mths, 6-7 mths, 9 mths, 1 yr, 5 yrs | 1 yr |
| Norway-Alabama | N | N | N | A always: 14-18, 22-26, 30-34, 36-38 weeks  B  P: hospital discharge, 1 mth, 6-7 mths, 1 yr, 5-6 yrs | 1 or 2 yrs |

HR = Heart rate. HR react = Heart rate reactivity. A = Antenatal. B = Birth. P = Postnatal. Y = Yes. N = No.

^a^ The retention rate for neurodevelopmental follow-up is around 91% at the UCLH site [5], was 90.5% for the ePrime dataset (with no difference between FGR-EO vs. controls [4], was 85% for the Alabama dataset with a slightly higher rate for SGA, FGR or FGR-EO (87%) vs. controls, and is not available for the Norway dataset. (71% of the Norway dataset received follow-up but loss to follow-up was due to either families not being invited at the time of delivery, or being invited but declined, and separate figures for the two reasons are unobtainable. The perinatal characteristics of Norway subjects did not differ between those who received and did not receive follow-up.)

**Supplementary to Methods/*Subjects*: When FGR was dissociated from SGA without Doppler measurements, FGR classification was associated with markers of placental insufficiency**

Dissociation of FGR from SGA could be inaccurate in the absence of Doppler information. Four data sources contributed both FGR and SGA subjects: in the neuro and FEMINA2 data, FGR/FGR-EO was dissociated from SGA using Doppler measurements; in the Norway-Alabama dataset, FGR/FGR-EO was dissociated from SGA using evidence of antenatal adversity associated with placental insufficiency (e.g. maternal preeclampsia) (Table S1). Given that the Norway-Alabama dataset was the only one to dissociate FGR from SGA without Doppler measurements, we reviewed those subjects’ placental data - when available - to confirm that FGR classification was associated with markers of placental insufficiency.

Although placental abnormalities are not reliably present in FGR [6], while surprisingly common in normal pregnancies [7], higher occurrence of placental hypoplasia (placental weight <10^th^ centile) and/or non-peripheral placental infarction in FGR/FGR-EO relative to SGA and controls would support that assignment to categories was sensible [8]. Placental weight and infarction information was available for 62-63 FGR (12 FGR-EO, 50-51 FGR), 19-20 SGA, and 401-403 control subjects respectively in the Norway-Alabama dataset. After accounting for sex and weeks since conception at delivery, placental weight was <10^th^ centile in 13/401 (3%), 1/19 (5%), 10/50 (20%) and 3/12 (25%) of control, SGA, FGR, and FGR-EO subjects respectively (chi-squared p = 0.0005). (Note that we calculated the distribution of placental weights separately for the two sites to identify the 10^th^ centile threshold, to account for possible differences in how the placentas had been prepared). One or more non-peripheral placental infarcts were observed in 25/403 (6%), 0/20 (0%), 7/51 (14%) and 1/12 (8%) of control, SGA, FGR, and FGR-EO subjects respectively (n.s.). Pooling both measures, the placenta had weight <10^th^ centile or/and non-peripheral infarcts in 39/403 (10%), 1/20 (5%), 13/51 (26%) and 4/12 (33%) control, SGA, FGR, and FGR-EO subjects respectively (chi-squared p = 0.0015).

In sum then, reassuringly, classification based on antenatal adversity associated with placental insufficiency (e.g. maternal preeclampsia) mapped onto independent (placental) biomarkers, supporting that assignment to FGR vs. SGA was reasonable, even when Doppler information was not available.

**Supplementary to Heart rate****: Postnatally, lower weeks since conception at birth predicted higher heart rate**

We separately analysed the subset of heart rate at rest data acquired postnatally, between 23 and 92 weeks since conception (total 137 subjects, 195 measures; FGR-EO: 73 subjects, 130 measures; FGR: 18 subjects, 19 measures; SGA and controls: 3 and 43 (no repeated measures)). The proportions calculated from transcutaneous pulse oximetry:ECG recordings were 151:44 (77%:23%). Using this postnatal data, we could examine the effect on heart rate of weeks since conception at birth (range 23-41 weeks).

Across subjects, for every 1 fewer week since conception at birth, heart rate was 0.9 beats per minute higher (t = 2.871), in line with previous work [9,10]. In this smaller sample, there was a non-significant trend for FGR-EO subjects to have a higher heart rate than controls (mean 148.4 vs. 143.8, t = 1.879), and weeks since conception at measure was not predictive but the direction of its coefficient was in the same direction as described in the main results. We checked that method of heart rate calculation did not predict heart rate (AIC worsened from 1557.3 to 1559.2).

**Supplementary to FGR-EO infants had smaller relative white matter volume: sensitivity analysis re. influence of multiple pregnancies**

**Singleton FGR-EO infants had smaller relative white matter volume**

After excluding multiple gestation subjects, structural MRI data were available from 185 subjects (37 FGR-EO; 148 controls). White matter volume was 1.0 cm^3^ higher/week since conception at birth (t = 2.812), 0.2 cm^3^ higher/1 cm^3^ total intracranial volume (t = 10.417), and 3.0 cm^3^ lower/week since conception at MRI (t = -4.789; as white matter volume was normalised by total intracranial volume in this model, this reflects its *relative* developmental decline: see Fig. 13 in [11]). FGR-EO subjects had mean 5 cm^3^ lower white matter volume than controls (mean 119.7 vs. 125.1 cm^3^, t = -2.666).

**Supplementary to** **Body weight across development: visualisation of model**


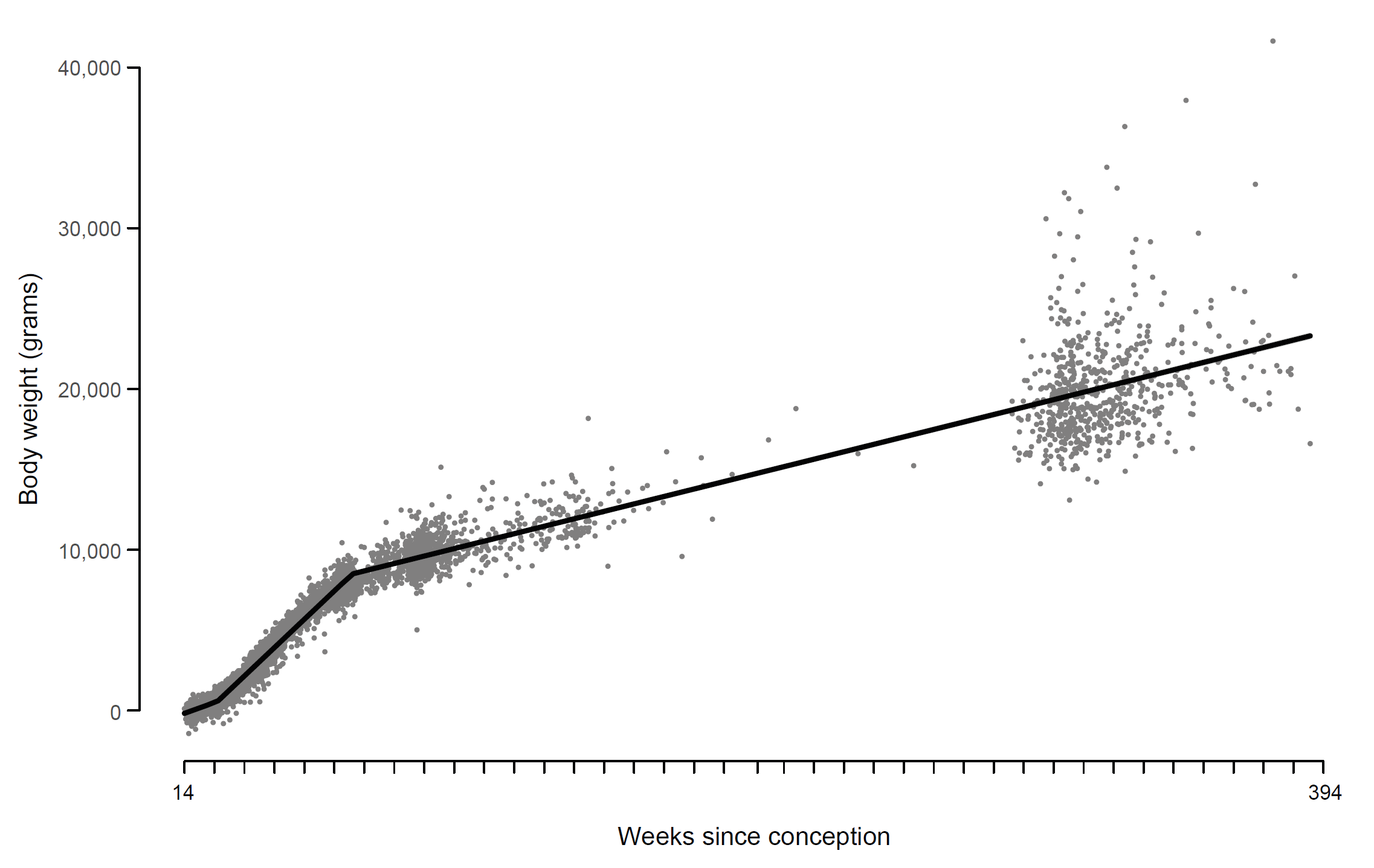


**Fig. S1: Piecewise regression model of the effect of weeks since conception on body weight.** Note the two knots (breakpoints) at 25 and 70 weeks. Figure created using R package visreg [12].

**Supplementary to Body weight across development: sensitivity analysis re. influence of multiple pregnancies**

After excluding multiple gestation subjects, 1514 singletons had total 7483 body weight measures (antenatal:postnatal 2784:4699 (37%:63%)) (FGR-EO: 224 subjects, 1497 measures (47% antenatal); FGR: 98 subjects, 582 measures (40% antenatal); SGA: 300 subjects, 1183 measures (5.2% antenatal); controls: 892 subjects, 4221 measures (42% antenatal)).

**Singleton FGR subjects continued to have lower body weight to six years of age**

Between 25-70 weeks since conception, FGR-EO and FGR subjects were mean 2155 and 1234 grams lighter than controls (t = -6.642, -3.872; SGA n.s.). Between 70-390 weeks since conception, FGR-EO and FGR subjects were mean 2802 and 1714 grams lighter than controls (t = -6.219, -4.640; SGA n.s.). Next, we tested whether - accounting for their lower weight at the onset of each piece – FGR-EO, FGR and SGA subjects had lower weight *gain* than controls. Between 25-70 weeks since conception, FGR-EO and FGR subjects gained mean 32 and 16 fewer grams/week of weight than controls (t = -12.250, -4.104; SGA n.s.). Between 70-390 weeks since conception, SGA subjects gained mean 2 fewer grams/week of weight than controls (t = -4.245; FGR and FGR-EO n.s.). Finally, we examined whether group affected the change in slope at 25 and 70 weeks since conception. Relative to controls, the change in slope at 70 weeks since conception was +30 and +15 grams/week in FGR-EO and FGR (t = 9.149 and 3.308; SGA n.s.) (25 weeks: all n.s.).

**Supplementary to** **Neurodevelopmental outcomes: sensitivity analysis re. influence of multiple pregnancies**

**Prematurity, FGR, and poorer individual growth,** **were associated with lower motor score in singletons**

A motor score was available for 915 singletons, born between 23 and 43 weeks since conception (of these: 561 (61%) controls 24-43 weeks; 190 (21%) SGA 29-43 weeks, 58 (6%) FGR 33-42 weeks, 106 (12%) FGR-EO 23-41 weeks). Motor score was 1 point lower/1 fewer week since conception at birth (t = -8.003). It was 6, 5, and 3 points lower in FGR-EO, FGR, and SGA relative to controls (mean 106, 107, and 109 relative to 112, t = -3.535, -2.530, -2.375). Motor score was also lower with more negative growth random effect: 0.4 points lower/-100 grams (t = -3.472).

**Prematurity, FGR-EO, and** **poorer individual growth in FGR-EO subjects, were associated with lower cognitive score in singletons**

A cognitive score was available for 921 singletons (almost the same demographics as for motor score). We included an interaction term between group and growth random effect (AIC improved from 6765.3 to 6755.8). Cognitive score was 0.9 points lower/1 fewer week since conception at birth (t = -7.531). It was lower in FGR-EO (but not FGR or SGA) relative to controls (mean 106 vs. 111, t = -3.388). Cognitive score was not overall associated with growth random effect, but this interacted with FGR-EO (extra 1.8 points lower/-100 grams in FGR-EO relative to controls, t = -3.939).

**Supplementary to Discussion: There is no evidence from existing literature that nutritional practices notably impact growth or neurodevelopment when other factors are adjusted for**

First examining SGA and FGR infants alone, there is little evidence that breast vs. formula milk significantly impacts their growth [13,14]. The effect sizes of feeding practices on body weight in this population are nil/n.s. (e.g. 4 months: 6.3 vs. 6.4 kg [15]; 2 years: 10.7 kg vs. 10.7 kg [16]) to small (e.g. 3 years: 1.31 kg vs. 1.28 kg according to whether any breastfeeding at hospital discharge [17]; 5-8 years: 8.3 kg vs. 8.2-8.5 kg according to whether formula was non-nutrient enriched or nutrient-enriched [18]). After adjusting for the association of breastfeeding with higher socio-economic and educational background, breast feeding does not predict higher motor or cognitive Bayley score in SGA infants [19].

Differences in nutritional practices also only co-vary modestly with our variable of interest (controls, SGA, FGR, FGR-EO) [20,21]. For example, 17% of infants ≤10^th^ birth weight centile and 29% of controls were exclusively breastfed at 16 weeks of age in [19]. Further, there is no notable interaction effect between nutritional practices and SGA/FGR vs. controls: nutritional interventions are comparably effective in SGA vs. control infants [22].

In sum, any differences in feeding practices across the infants in this study are unlikely to explain the effect sizes of difference observed here between groups.

**Bibliography**

1. Omole, O., Palin, V., Watson, K. & Myers, J. Examining the Magnitude of Maternal Ethnic and Socioeconomic Inequalities on Foetal Growth Restriction and Preterm Birth: A Cohort Study Set in North West England. J. Racial Ethn. Health Disparities https://doi.org/10.1007/s40615-025-02437-2 (2025) doi:10.1007/s40615-025-02437-2.

2. Dongarwar, D., Garcia, B. Y., Booker, A., Sankhavaram, M. & Salihu, H. M. Implications of Social Determinants of Health Characteristics on Fetal Growth Restriction Among Various Racial/Ethnic Groups. Matern. Child Health J. 27, 650–658 (2023).

3. Higgins, L. E., Myers, J. E., Sibley, C. P., Johnstone, E. D. & Heazell, A. E. P. Antenatal placental assessment in the prediction of adverse pregnancy outcome after reduced fetal movement. PLOS ONE 13, e0206533 (2018).

4. Sacchi, C. et al. Neurodevelopmental Outcomes following Intrauterine Growth Restriction and Very Preterm Birth. J. Pediatr. 238, 135-144.e10 (2021).

5. Durrant, C. et al. Developmental trajectories of infants born at less than 30 weeks’ gestation on the Bayley-III Scales. Arch. Dis. Child. - Fetal Neonatal Ed. 105, 623–627 (2020).

6. Barak, O. et al. Integrated unbiased multiomics defines disease-independent placental clusters in common obstetrical syndromes. BMC Med. 21, 349 (2023).

7. Romero, R. et al. The frequency and type of placental histologic lesions in term pregnancies with normal outcome. J. Perinat. Med. 46, 613–630 (2018).

8. Khong, T. Y. et al. Sampling and Definitions of Placental Lesions: Amsterdam Placental Workshop Group Consensus Statement. Arch. Pathol. Lab. Med. 140, 698–713 (2016).

9. Burtchen, N. et al. Autonomic signatures of late preterm, early term, and full term neonates during early postnatal life. Early Hum. Dev. 137, 104817 (2019).

10. Aye, C. Y. L. et al. Neonatal autonomic function after pregnancy complications and early cardiovascular development. Pediatr. Res. 84, 85–91 (2018).

11. Makropoulos, A. et al. Regional growth and atlasing of the developing human brain. NeuroImage 125, 456–478 (2016).

12. Breheny, P. & Burchett, W. Visualization of Regression Models Using visreg. R J. 9, 56–71 (2017).

13. Atzemoglou, N. et al. Does Breastfeeding Small for Gestational Age Neonates Promote a Healthier Growth Pattern? A Narrative Review. Children 12, 1227 (2025).

14. Santiago, A. C. T. et al. Breastfeeding in children born small for gestational age and future nutritional and metabolic outcomes: a systematic review. J. Pediatr. (Rio J.) 95, 264–274 (2019).

15. de Zegher, F. et al. Body Composition and Circulating High-Molecular-Weight Adiponectin and IGF-I in Infants Born Small for Gestational Age : Breast- Versus Formula-Feeding. Diabetes 61, 1969–1973 (2012).

16. Hofi, L., Flidel-Rimon, O., Hershkovich–Shporen, C., Zaharoni, H. & Birk, R. Differences in growth patterns and catch-up growth of small for gestational age preterm infants fed on fortified mother’s own milk v. preterm formula. Br. J. Nutr. 129, 2046–2053 (2023).

17. Vizzari, G. et al. Postnatal growth of small for gestational age late preterm infants: determinants of catch-up growth. Pediatr. Res. 94, 365–370 (2023).

18. Singhal, A. et al. Nutrition in infancy and long-term risk of obesity: evidence from 2 randomized controlled trials. Am. J. Clin. Nutr. 92, 1133–1144 (2010).

19. Rao, M., Hediger, M., Levine, R., Naficy, A. & Vik, T. Effect of breastfeeding on cognitive development of infants born small for gestational age. Acta Paediatr. 91, 267–274 (2002).

20. Toftlund, L. H., Halken, S., Agertoft, L. & Zachariassen, G. Catch-Up Growth, Rapid Weight Growth, and Continuous Growth from Birth to 6 Years of Age in Very-Preterm-Born Children. Neonatology 114, 285–293 (2018).

21. Lingam, I. et al. Neonatal outcomes following early fetal growth restriction: a subgroup analysis of the EVERREST study. Arch. Dis. Child. - Fetal Neonatal Ed. https://doi.org/10.1136/archdischild-2022-325285 (2023) doi:10.1136/archdischild-2022-325285.

22. Roggero, P. et al. Implementation of Nutritional Strategies Decreases Postnatal Growth Restriction in Preterm Infants. PLOS ONE 7, e51166 (2012).
